# In Situ Fabrication of Silver Nanoparticle‐Decorated Polymeric Vesicles for Antibacterial Applications

**DOI:** 10.1002/open.202300223

**Published:** 2024-04-22

**Authors:** Fen Zhang, Qian Yao, Yanling Niu, Xiaoqi Chen, Haijun Zhou, Lu Bai, Zejuan Kong, Yantao Li, Hua Cheng

**Affiliations:** ^1^ Institute of Energy Resources Hebei Academy of Sciences 050081 Shijiazhuang Hebei Province China; ^2^ Institute of Biology Hebei Academy of Sciences 050081 Shijiazhuang Hebei Province China

**Keywords:** silver nanoparticles, polymeric vesicles, redox-initiated, RAFT dispersion polymerization, antibacterial properties

## Abstract

Silver/polymeric vesicle composite nanoparticles with good antibacterial properties were fabricated in this study. Silver nanoparticles (AgNPs) were prepared in situ on cross‐linked vesicle membranes through the reduction of silver nitrate (AgNO_3_) using polyvinylpyrrolidone (PVP) via coordination bonding between the Ag^+^ ions and the nitrogen atoms on the vesicles. X‐ray diffraction (XRD), ultraviolet‐visible spectroscopy (UV‐vis), and transmission electron microscopy (TEM) analyses confirmed the formation of AgNPs on the vesicles. The antibacterial test demonstrated good antibacterial activity against both Gram‐negative bacteria (*Escherichia coli*) and Gram‐positive bacteria (*Staphylococcus aureus*) for the produced AgNP‐decorated vesicles. The minimum inhibitory concentration (MIC) values of the AgNP‐decorated vesicles for *E. coli* and *S. aureus* were 8.4 and 9.6 μg/mL, respectively. Cell viability analysis on the A549 cells indicated that the toxicity was low when the AgNP concentrations did not exceed the MIC values, and the wound healing test confirmed the good antibacterial properties of the AgNP‐decorated vesicles.

## Graphical abstract

In this study, cross‐linked vesicles with nitrogen atoms are used as templates for the fabrication of silver nanoparticle (AgNP)‐decorated vesicles. AgNPs are prepared in situ on the vesicle membrane by the reduction of silver nitrate (AgNO_3_) using polyvinylpyrrolidone (PVP), and the produced AgNP‐decorated vesicles exhibit good antibacterial activity against both E. coli and S. aureus.

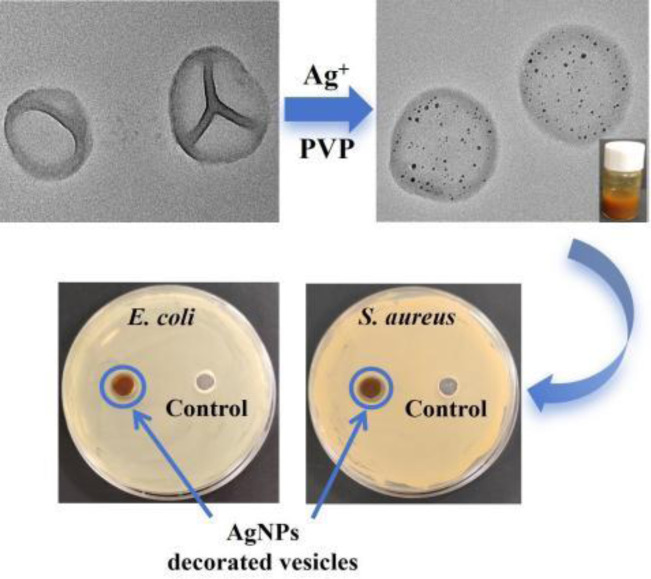

## Introduction

Infectious diseases, which pose a significant threat to human life, have emerged as a major global health concern. The outbreak of COVID‐19 in 2019 has drawn increasing attention to the treatment of bacterial and viral infections.[^1^, ^2^] However, the overuse of antibiotics has led to drug resistance and the development of more bacterial pathogens, making it difficult to treat infectious diseases.^[3]^ Therefore, there is a pressing need to develop new antibacterial agents that do not contribute to drug resistance, providing an alternative to conventional antibiotics.

Nanoparticles have been used for antibacterial applications, as they do not generate drug resistance and they increase antibiotic interactions with bacteria and viruses due to their special nano‐scale characteristics. Many studies have been conducted to improve the antibacterial properties of nanoparticles. Safaei et al. studied the synthesis and properties of alginate‐manganese oxide bionanocomposites, polyhydroxybutyrate Co_3_O_4_ bionanocomposites, and sodium hyaluronate‐TiO_2_ bionanocomposites.[^4^, ^5^, ^6^] By optimizing synthesis conditions, three types of nanocomposite, all possessing high antibacterial activity, could be prepared. In addition, silver nanoparticles (AgNPs) have been widely studied and used, especially compared to other antibacterial materials such as quaternary ammonium moieties, silica‐based materials, and antimicrobial peptides,[^7^, ^8^, ^9^, ^10^, ^11^, ^12^] due to their long‐lasting and broad‐spectrum antibacterial properties, which do not engender drug resistance. However, the tendency of AgNPs to agglomerate seriously restricts their application,[^13^, ^14^] leading to a decline in their antibacterial efficacy when they aggregate into larger particles.^[15]^ Polymeric templates such as polymer chains, micelles, or vesicles can effectively prevent AgNP aggregation, and self‐assembled nanostructures can significantly improve antibacterial efficacy for the increased local concentration of well‐dispersed AgNPs.[^14^, ^16^, ^17^]

Polymeric vesicles, due to their hollow inner cavity and significantly higher specific surface areas than other nano‐scale templates, have proven to be ideal carriers for drug delivery and therapeutic applications.[^18^, ^19^] Du et al. studied the antibacterial performance of AgNP‐decorated polymeric vesicles and observed that these AgNPs exhibited good stability, with the AgNP‐decorated polymeric vesicles showing excellent antibacterial properties.[^17^, ^20^, ^21^] The polymeric vesicles served as a reservoir for the AgNPs, gradually releasing Ag^+^ ions to restrain bacterial growth,^[22]^ prolonging the inhibition time, and decreasing cytotoxicity. However, the vesicles were prepared by a conventional self‐assembly method, with a complicated production process, and a general low solid content of less than 1 wt %.[^23^, ^24^]

Polymerization‐induced self‐assembly (PISA) has gained interest as a method to prepare polymeric nanoparticles, due to its high efficiency (combination of one‐pot polymerization and self‐assembly), high solid content, better designability, and ability to prepare polymeric vesicles with high monomer conversion.[^25^, ^26^, ^27^, ^28^, ^29^] By introducing functional monomers, the cross‐linking of the vesicle membrane can be realized during polymerization or through post‐polymerization strategies. This can further improve the stability of the vesicles, making them suitable for use in acidic, alkaline, high shearing, and high dilution environments, as well as in situations involving the addition of a solvent for both blocks.^[30]^ Consequently, these vesicles have become more attractive as templates for AgNP growth.

In this study, a facile method for preparing antibacterial AgNP‐decorated vesicles is reported. By using cross‐linked polymeric vesicles as the template, AgNPs were fabricated in situ on vesicle membranes via the reduction of AgNO_3_ using PVP through coordination bonds between the Ag^+^ ions and nitrogen atoms on the vesicles. The vesicles were produced by redox‐initiated reversible addition‐fragmentation chain transfer (RAFT) dispersion polymerization with a 2‐(diisopropylamino)ethyl methacrylate (DIPEMA) and glycidyl methacrylate (GlyMA) in an ethanol–water mixture, and the vesicle membranes were cross‐linked through the post‐polymerization of epoxydiamine chemistry, as reported in our previous study.^[31]^ The PDIPEMA blocks on the vesicles allowed the nitrogen atoms to coordinate with the Ag^+^ ions, and cross‐linking of the vesicle membrane initially improved vesicle stability and introduced more amine groups, improving the attachment ability with Ag^+^ ions.[^17^, ^32^, ^33^] Subsequently, XRD, UV‐vis, and TEM analyses were applied to investigate the formation of AgNP‐decorated vesicles, and their antibacterial properties were evaluated.

## Results and Discussion

### Preparation and Characterization of the AgNP‐Decorated Vesicles

**Figure 1:**
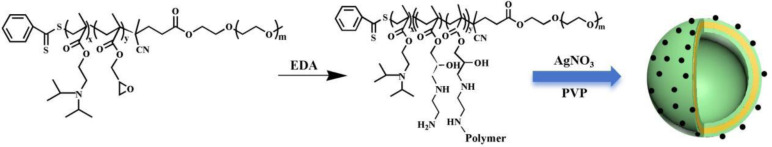
Chemical reaction and structural diagram of the produced polymer.

Inorganic/organic composite materials combine the advantages of inorganic and organic materials, endowing the materials with unexpected properties. The deposition of inorganic nanoparticles onto polymeric nanoparticles serves as a common approach to prepare inorganic/organic composite materials.[^34^, ^35^] In this study, the in situ deposition of AgNPs on polymeric vesicles was conducted using cross‐linked polymeric vesicles as templates. Polymeric vesicles mPEG‐b‐P(DIPEMA‐co‐GlyMA) were produced through redox‐initiated RAFT dispersion polymerization, as previously reported.^[31]^ The cross‐linking of the vesicle membrane was conducted using ethylenediamine (EDA) to react with the epoxy groups on the vesicles, to improve the stability. In addition, a certain number of amine groups were further introduced into the vesicles, as reported by Tan et al.^[32]^ for poly(glycerol monomethacrylate)_46_‐b‐poly(2‐hydroxypropyl methacrylate)_300_‐bpoly(glycidyl methacrylate)_300_ vesicles, where free amine groups were introduced through epoxydiamine chemistry. Using coordination bonds between the Ag^+^ ions and nitrogen atoms on the vesicle membranes, the AgNPs could deposit in situ on the cross‐linked vesicles. The chemical reaction is shown in Figure 1.

**Figure 2:**
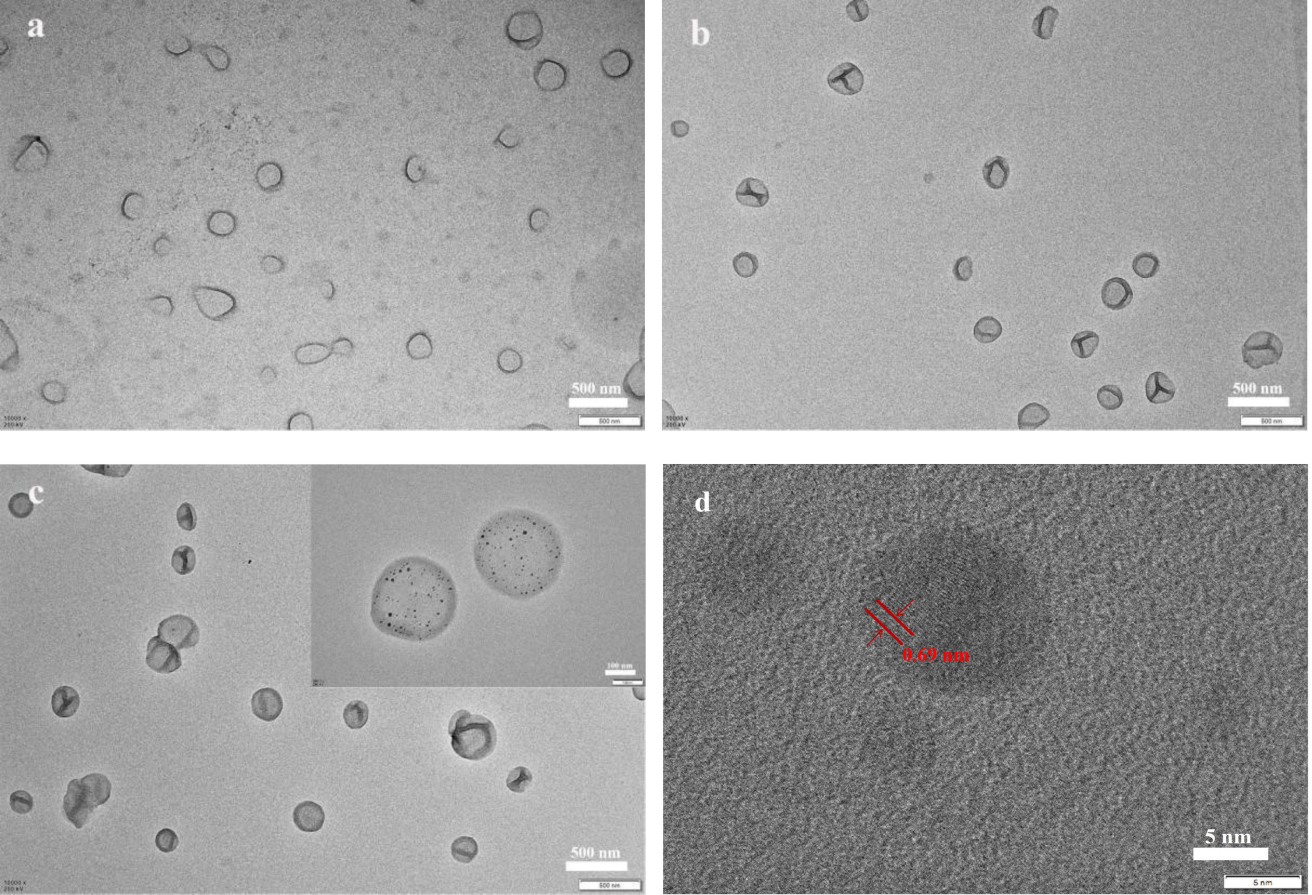
(a) TEM image of the polymeric vesicles prepared by redox‐initiated RAFT dispersion polymerization, (b) TEM image of polymeric vesicles after reaction with EDA, (c) TEM image of the AgNP‐decorated vesicles prepared by the in situ reduction of AgNO_3_, and (d) TEM image of the AgNPs on the vesicles.

For the preparation of the cross‐linked vesicle templates, non‐cross‐linked vesicles were first fabricated using DIPEMA and GlyMA (DIPEMA/GlyMA molar ratio=8 : 2), which copolymerized in ethanol‐water mixtures with 40 wt % water via redox‐initiated RAFT dispersion polymerization. The morphology is shown in Figure 2 (a). An excessive amount of EDA was added to the diluted vesicle solution to cross‐link the vesicle membranes, and the degree of cross‐linking was about 91 %, calculated by weight. After purification through centrifugation‐redispersion cycles, cross‐linked vesicles with a well‐defined shape could be prepared, as shown in Figure 2 (b).

To prepare the AgNP‐decorated vesicles, the cross‐linked vesicles were dispersed in ethanol‐water mixtures, and the solution was stirred for 30 min in the dark after AgNO_3_ addition. After PVP was added, the dispersion gradually turned from very light yellow to brownish in color, indicating AgNP formation. The morphology of the cross‐linked vesicles after AgNP deposition was characterized by TEM, as shown in Figure 2 (c), where small black nanoparticles in the range of 5–12 nm in size could be observed on the vesicle membranes. Lattice fringes with a spacing of 0.23 nm were observed in the TEM image (Figure 2 (d)) for the small black nanoparticles, which was consistent with previously reported crystalline AgNP structures.[^36^, ^37^] The above results indicated that the AgNPs were synthesized in situ on the cross‐linked vesicles.

**Figure 3:**
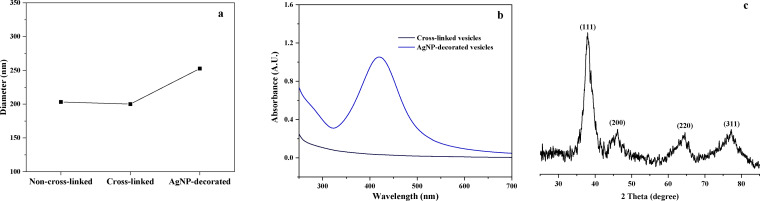
(a) Particle size of non‐cross‐linked, cross‐linked and AgNP‐decorated vesicles, (b) UV‐vis spectra of the dispersions of cross‐linked vesicles and the AgNP‐decorated vesicles, and (c) XRD spectra of the AgNP‐decorated vesicles.

Changes in the particle size of non‐cross‐linked, cross‐linked, and AgNP‐decorated vesicles were also investigated through dynamic light scattering (DLS), as shown in Figure 3 (a). The particle size did not exhibit significant changes before and after cross‐linking, remaining at about 200 nm in diameter (they changed from 203 nm to 198 nm, so the cross‐linking of the vesicle membrane caused a slight shrinkage in the vesicle). However, a small increase could be observed for the AgNP‐decorated vesicles, where the AgNPs deposited on the vesicle surface caused an increase in particle size.

To further confirm the formation of the AgNPs, UV‐vis, and XRD analyses were performed to characterize the AgNP‐decorated vesicle samples. Figure 3 (b) shows the UV‐vis spectra before and after AgNP decoration. A sharp peak at approximately 415 nm was observed for the vesicles after decoration with AgNPs, corresponding to the surface plasmon resonance of the AgNPs (the characteristic peak of the AgNPs was in the range of 400–420 nm), while no noticeable absorbance was observed for the pure vesicle samples.[^21^, ^38^] In addition, diffraction peaks in the XRD results (Figure 3(c)) were located at 38.0°, 45.9°, 64.5°, and 77.2°, which corresponded to the (111), (200), (220), and (311) facets of the face‐centered cubic (FCC) AgNPs, respectively.[^35^, ^39^]

### Antibacterial Properties of the AgNP‐Decorated Vesicles

AgNPs have shown a wide variety of antibacterial properties, effectively killing both Gram‐negative and Gram‐positive bacteria, such as *E. coli* and *S. aureus*. As a result, AgNPs have been used in the medical field to treat infections, solving the issue of multi‐drug resistance.[^20^, ^22^, ^40^, ^41^] The antibacterial properties of the produced AgNP‐decorated vesicles were evaluated by the MIC method and inhibition zone method. The MIC method could quantitatively determine the minimum concentration of AgNPs at which bacterial growth could not be detected using optical density (OD) analysis, while the inhibition zone method could qualitatively evaluate antibacterial performance.

**Figure 4:**
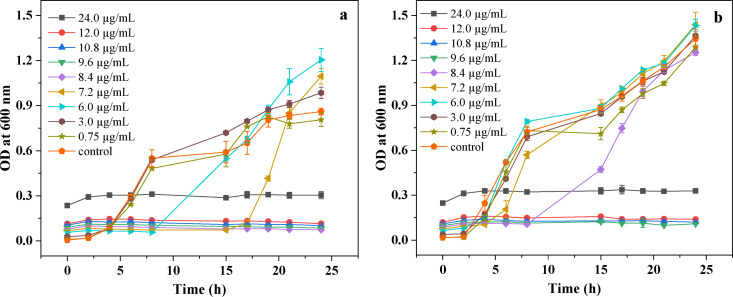
Time‐dependent growth curves of (a) *E. coli* and (b) *S. aureus* with different AgNP concentrations. Pure cross‐linked vesicles were used as the control.

For the MIC study, a series of AgNP‐decorated vesicle solutions was prepared by dilution with the culture medium, using atomic absorption spectroscopy (AAS) to evaluate the concentration of AgNPs. Pure cross‐linked vesicles were used as the control sample to eliminate the effect of the polymeric vesicles. Subsequently, each sample and bacterial dispersion was poured into the culture tube for incubation, and during the test, the ODs of the samples were assessed at different incubation times. Figure 4 presents the time‐dependent growth curves of (a) *E. coli* and (b) *S. aureus* with different AgNP concentrations within 24 h. As shown in Figure 4(a), no *E. coli* growth was observed for samples with AgNP concentrations equal to or greater than 8.4 μg/mL. With a further decrease in AgNP concentration, the inhibition time for *E. coli* decreased, and when the AgNP concentration decreased to 3.0 μg/mL, the OD value demonstrated a noticeable increase after 4 h of incubation, similar to the control sample pure vesicles. This indicated that the antibacterial properties were very weak at this concentration. A similar inhibition curve was observed for *S. aureus*, as shown in Figure 4(b), where no microbial growth was found for the samples with AgNP concentrations equal to or greater than 9.6 μg/mL. When the AgNP concentration decreased to 6.0 μg/mL, the OD curve was similar to the control sample, and after incubation for 2 h, the OD value started to increase. The MIC values of the AgNPs for *E. coli* and *S. aureus* were 8.4 and 9.6 μg/mL respectively, and these values were in the reported MIC range of 5–10 μg/mL.^[17]^ A slightly better antibacterial property against *E. coli* was found, which was consistent with the reported results, and this difference might be attributed to the cell wall structure and compositions of the bacteria. The rigid thick peptidoglycan layer of *S. aureus* may prevent the entry of AgNPs, explaining the observed variation.[^42^, ^43^]

**Figure 5:**
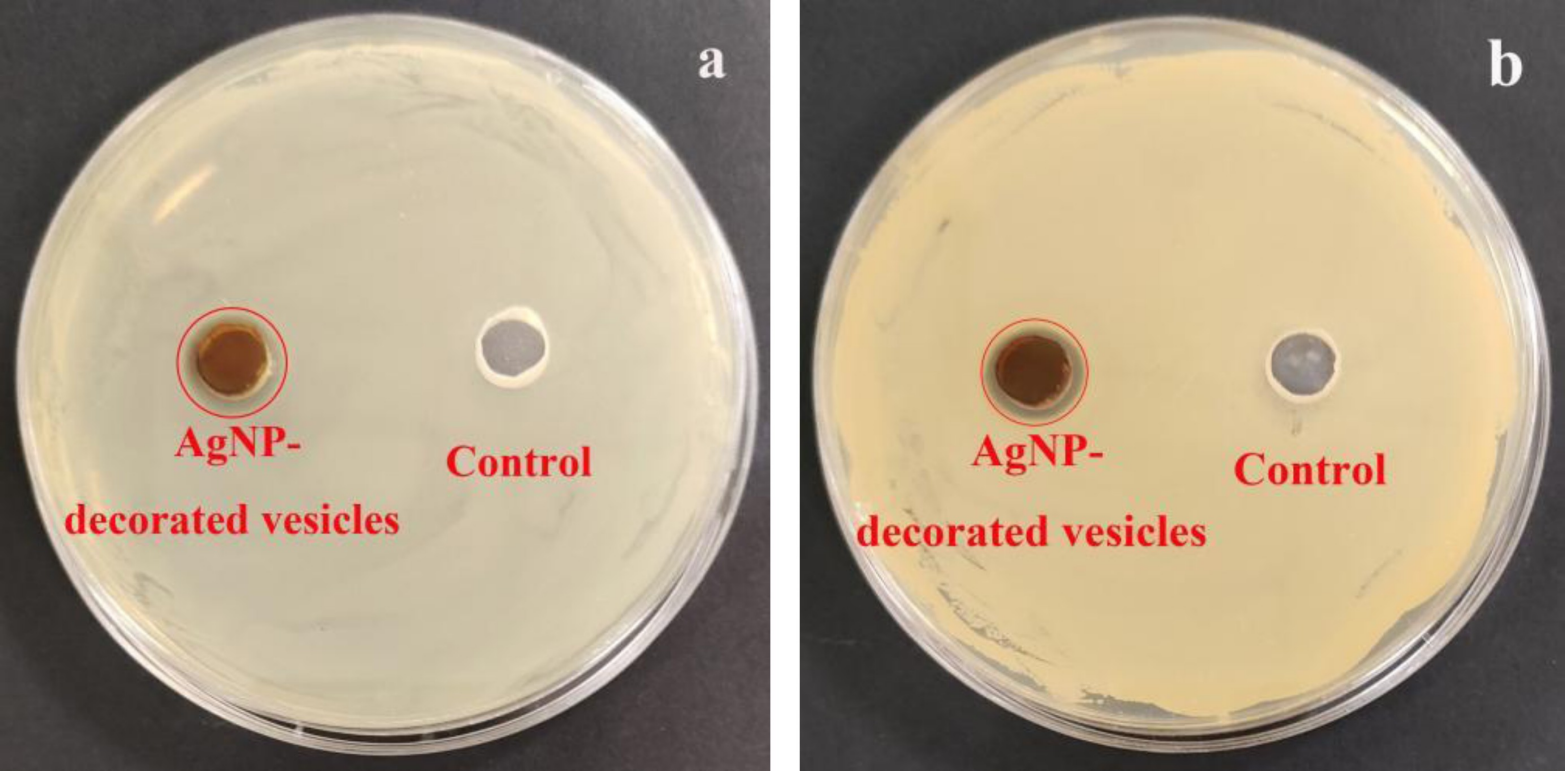
Inhibition zones for the AgNP‐decorated vesicles and control sample pure cross‐linked vesicles against (a) *E. coli* and (b) *S. aureus* after incubation for 18 h.

The inhibition zones of the AgNP‐decorated vesicles against *E. coli* and *S. aureus* were also studied, and pure cross‐linked vesicles were used as the control sample, as shown in Figure 5.For the control sample, no inhibition zones were observed for both *E. coli* and *S. aureus*, while clear inhibition zones were found for the AgNP‐decorated vesicles. The diameters of the inhibition zones for *E. coli* and *S. aureus* were both ~2 mm, indicating good antibacterial properties of the AgNP‐decorated vesicles against both *E. coli* and *S. aureus* bacteria.

### Cell Viability Test

**Figure 6:**
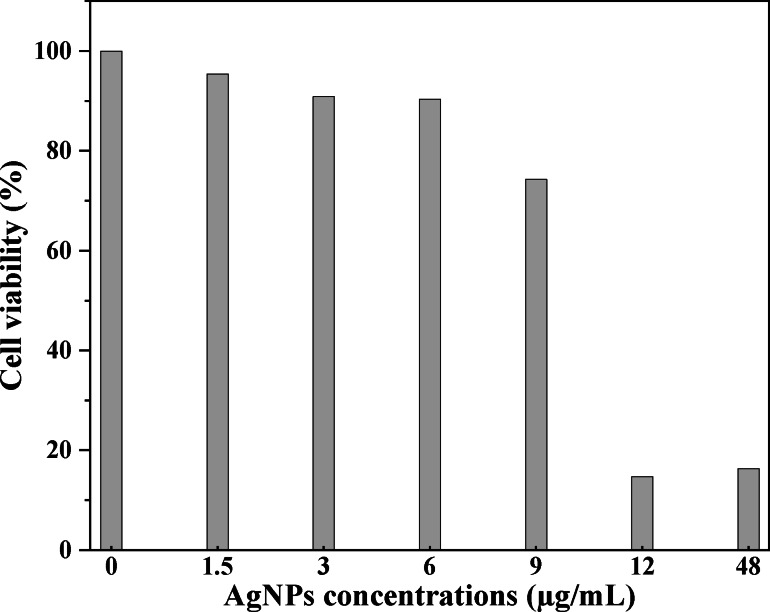
Cytotoxicity of the AgNP‐decorated vesicles after 24 h of incubation with the A549 cells.

A cell counting kit‐8 (CCK‐8) assay was used for the cell viability test of the AgNP‐decorated vesicles on the A549 cells. A series of AgNP‐decorated vesicle solutions was prepared using the cell culture medium and added to the pre‐incubated cells, with pure cross‐linked vesicles used as the control. After incubation and treatment with CCK‐8 solution, the absorbance values of the samples were tested for cell viability calculation, and Figure 6 shows the analysis results. We observed that the cell viability decreased with increasing AgNP concentration, and the cell viability was higher than 90 % when the AgNP concentration was below 9 μg/mL. A sharp decrease in cell viability was found with an additional increase in AgNP concentration from 9 to 12 μg/mL, indicating that the cytotoxicity of the AgNP‐decorated vesicles was significantly higher for the A549 cells at this concentration. The toxicity of the AgNPs was high and could destroy the structure of the bacterial cell membranes.[^22^, ^44^] However, the produced AgNP‐decorated vesicles were safe for the A549 cells within the concentrations of the MIC values for the AgNPs.

### Application of AgNP‐Decorated Vesicles to Wound Healing

**Figure 7:**
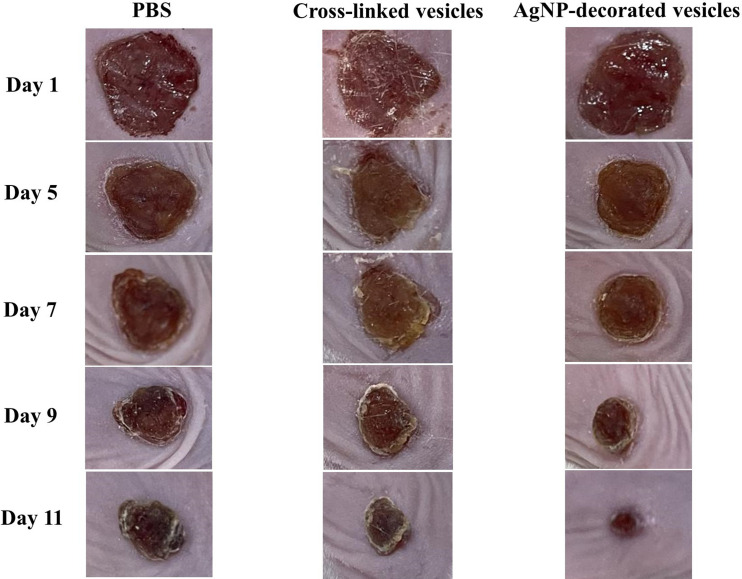
Images of the wound healing process for infected wounds treated with PBS, cross‐linked vesicles, and AgNP‐decorated vesicles.

**Figure 8:**
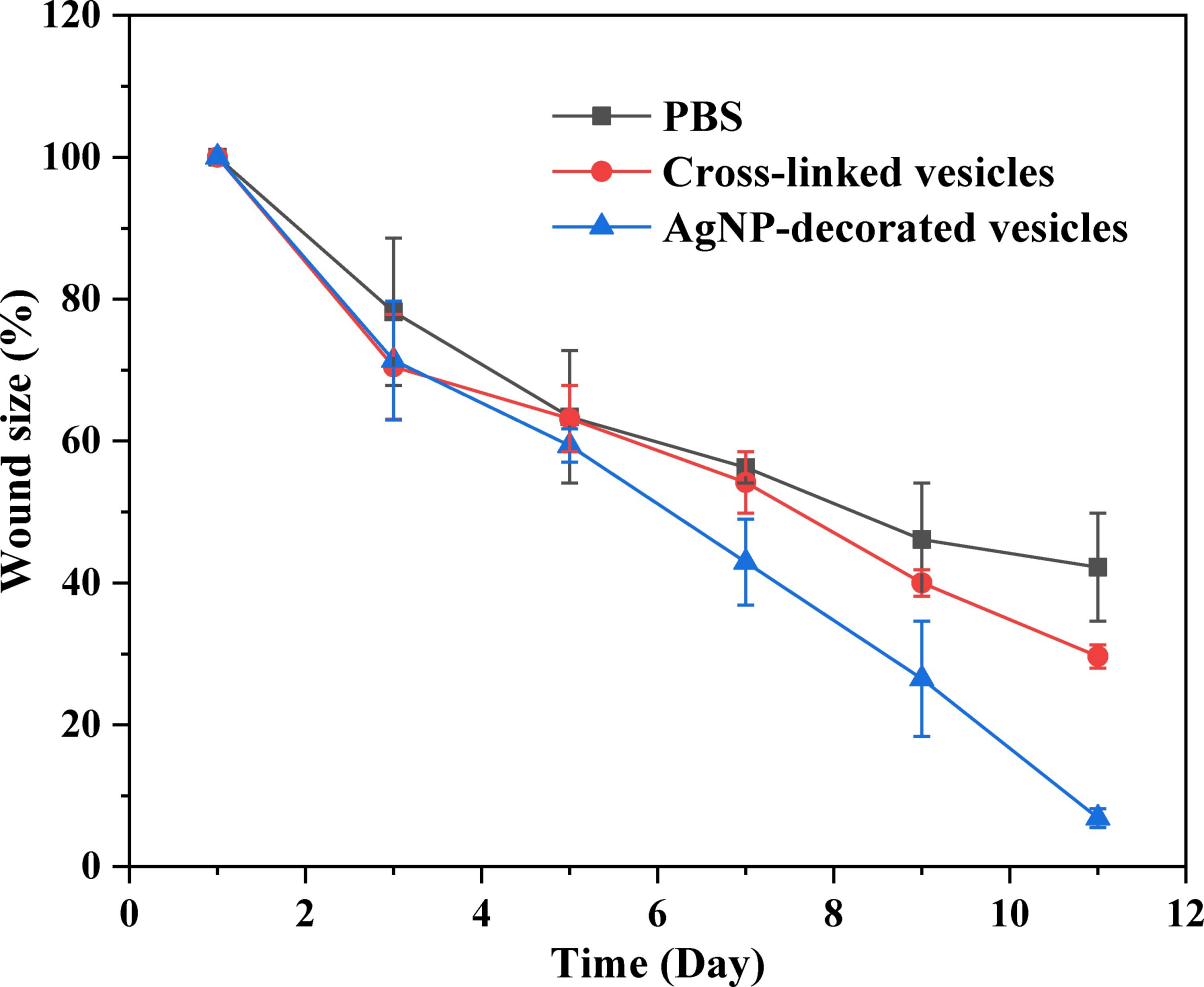
Trends of wound healing for the three groups of mice treated with PBS, polymeric vesicles, and AgNP‐decorated vesicles.

To evaluate the AgNP‐decorated vesicles in infected wound healing, full‐thickness skin wounds were created, and then *S. aureus* was spread on the wounds of the mice twice for infection. One day later, the wounds were treated with phosphate‐buffered saline (PBS), pure cross‐linked vesicle solution, and AgNP‐decorated vesicle solution, and this was recorded as day 1. Subsequently, the wounds were treated with three samples once per day for the next few days, and the wounds were continuously monitored for changes in size. Figure 7 shows the photos of the infected wound healing process treated with the three samples, and Figure 8 shows the trend of wound healing, which was conducted by checking the wound area. As shown in Figure 7, the wounds treated with AgNP‐decorated vesicles were almost healed after 11 days, which was significantly faster than the pure vesicles and PBS‐treated wounds, indicating that the AgNPs on the vesicles benefited infected wound healing and shortened the wound healing time. The wound healing rate was quantified by assessing the wound position area, by calculating the ratio of the wound area of each assessed day versus on day 1, as shown in Figure 8. Notably, the wounds treated with PBS and pure vesicles did not show significant differences, while the wounds treated with AgNP‐decorated vesicles demonstrated faster wound closure rates after day 7.

## Conclusions

In this study, cross‐linked vesicles with nitrogen atoms were used as templates for the preparation of AgNP‐decorated vesicles, exhibiting excellent antibacterial activity against both *E. coli* and *S. aureus*. The cross‐linking of the vesicle membranes improved stability and introduced more nitrogen atoms, which further improved attachment with the Ag^+^ ions, where AgNPs were successfully fabricated in situ on vesicle membranes through the reduction of AgNO_3_ using PVP. The MIC values of the AgNP‐decorated vesicles for *E. coli* and *S. aureus* were 8.4 and 9.6 μg/mL, respectively, and the toxicity was lower when the AgNP concentrations did not exceed the MIC values. This investigation afforded a facile method for preparing antibacterial AgNP‐decorated vesicles, which could potentially be used in wound healing applications.

## Experimental Section

### Materials

The α‐methoxy‐ω‐hydroxypoly(ethylene oxide) (mPEG), 4‐(4‐cyanopentanoic acid) dithiobenzoate (CPADB), 4‐(dimethylamino)pyridine, dicyclohexylcarbodiimide, and PVP compounds were purchased from Aladdin, and used as received. DIPEMA (Aladdin, 97 %) and GlyMA (Aladdin) were purified by passing the compounds through an Al_2_O_3_ column to remove the inhibitor before use. The redox‐initiator potassium persulfate (KPS, United Initiators [Shanghai] Co., Ltd.), as well as sodium bisulfite (SBS, Damao Chemical Reagent Factory) were used as received. AgNO_3_ was purchased from Tianjin Tiangan Chemical Technology Development Co., Ltd., and was also used as received. All other chemicals were of analytical grade and used without further purification.

### In Situ Generation of the AgNP‐Decorated Vesicles

The macro‐RAFT agent mPEG‐CPADB and the cross‐linked polymeric vesicles were prepared according to the procedures outlined in a previous study.^[31]^ DIPEMA (0.3072 g, 1.4400 mmol), GlyMA (0.0384 g, 0.3600 mmol), mPEG‐CPADB (0.0440 g, 0.0200 mmol), KPS (0.0019 g, 0.0067 mmol), SBS (0.0007 g, 0.0067 mmol), ethanol (2.1179 g, 0.0460 mmol), and water (1.4119 g, 0.0784 mmol) were added into a reaction tube containing a magnetic bar at a solid content of 10 wt %. After degassing through three pump‐N_2_ purge cycles, the tube was sealed and placed in an oven to react for 7 h at 35 °C under stirring. The reaction mixture was then quickly cooled in liquid nitrogen and open air to quench polymerization. The cross‐linking of the vesicles was conducted by using the above‐produced vesicles to react with EDA, and excess EDA was removed through centrifugation‐redispersion cycles.

The cross‐linked vesicles were subsequently used as templates to generate the AgNPs in situ. To obtain the AgNP‐decorated vesicles, the cross‐linked vesicles after centrifugation‐redispersion cycles were redispersed in ethanol/water solution (solid content of 0.002 g/mL, 5 mL), and then 0.0025 g of AgNO_3_ was added to the solution. After stirring for 30 min in the dark at room temperature, 0.0025 g of PVP was quickly added to the vesicle solution, and the reaction solution gradually became brown. After the reduction reaction proceeded for 24 h at 50 °C, the samples were purified through centrifugation‐redispersion cycles to remove any free silver particles in the solution.

### Characterization

The morphologies of the produced polymeric and AgNP‐decorated vesicles were analyzed through TEM (JEOL, JEM‐2100 Plus), and the samples were stained with phosphotungstic acid before observation. The particle diameter was determined using a DLS spectrometer (PPS, Z3000).

UV‐vis (Puxi, TU‐1901) and XRD (LabX, XRD‐6000) analyses were conducted to confirm the formation of the AgNPs, while AAS (Hitachi, Z‐2300) was used to assess the concentration of silver.

The antibacterial performance of the AgNP‐decorated vesicles was evaluated using the MIC and inhibition zone methods, and *E. coli* and *S. aureus* were used for the test. Preparation of the culture medium and culturing of the bacteria were conducted according to the standard method. For MIC analysis, the *E. coli* and *S. aureus* bacteria were cultured at 37 °C for 15 h and then diluted to approximately 10^7^ colony‐forming units (CFU)/mL for use, according to the reported literature.[^9^, ^21^] Different concentrations of AgNP‐decorated vesicle solutions were prepared through dilution with the culture medium, and 2 mL of each solution was filled into each culture tube, followed by the addition of 2 mL of bacterial dispersion. The pure cross‐linked vesicle solution was then used as the control sample. The tubes were incubated at 37 °C on a shaking bed, and samples were extracted at set times. The OD at 600 nm (OD_600_) was assessed using a Thermo Fisher 1510 instrument.

A qualitative study of the antibacterial performance of the AgNP‐decorated vesicles was conducted using the inhibition zone method. The nutrient agar was prepared according to the standard method, and pre‐incubated *E. coli* and *S. aureus* bacteria were used for evaluation. First, approximately 20 mL of fresh nutrient agar was added to each petri dish, and after solidification, 100 μL of bacterial suspension was added and evenly coated on each of the nutrient agar plates. Then, 9.5‐mm‐diameter wells were constructed on the agar plate, and 100 μL of sample solution was added to each well. Pure cross‐linked vesicle solution was used as the control sample. Then, the samples were placed into an incubator and incubated at 37 °C for 18 h. Colony growth was observed and photographed to measure the diameter of the inhibition zone, and the tests were carried out in triplicate.

The cytotoxicity of the AgNP‐decorated vesicles was analyzed by a CCK‐8 assay, with the study focusing on non‐small cell lung carcinoma A549 cells. For the evaluation, pre‐incubated A549 cells were filled into the 96‐well plates and incubated for 24 h at 37 °C. Then, the AgNP‐decorated vesicles with different concentrations were added to the wells and incubated with the cells for another 24 h. Later, the culture medium was removed and 100 μL of CCK‐8 solution was added. Then, the 96‐well plates were placed into the incubator for an additional 2 h. Finally, the samples were removed and the absorbance values were assessed on a Thermo 1510 instrument at a wavelength of 450 nm, and the values were used for cell viability calculations. Each sample was tested five times in parallel and the average value was recorded.

The in vivo antibacterial study was conducted using female BALB/c mice (~6 weeks) supplied by Hebei Medical University. During testing, each mouse was housed in an isolated cage and placed in the animal laboratory room. To produce a wound infection, hair on the back of the mice was removed and the skin was anesthetized. Then, wounds ~8 mm in diameter were created using a puncher. Subsequently, *S. aureus* with a concentration of 10^7^ CFU/mL was applied to the wounds twice, and the wounds were infected after 1 day. Pure cross‐linked vesicle solution, AgNP‐decorated vesicle solution, and PBS solution were used to treat the wound positions on the mice once per day. Photos were taken and changes in wound size were recorded every day for 11 days. The experiments were conducted simultaneously on three mice for each sample.

## Conflict of interests

The authors report no conflicts of interest in this work.

1

## Data Availability

Research data are not shared.
